# The Role of Attitudes, Norms, and Efficacy on Shifting COVID-19 Vaccine Intentions: A Longitudinal Study of COVID-19 Vaccination Intentions in New Zealand

**DOI:** 10.3390/vaccines9101132

**Published:** 2021-10-04

**Authors:** Jagadish Thaker, Somrita Ganchoudhuri

**Affiliations:** 1 School of Communication, Journalism & Marketing, Massey University, Wellington 6011, New Zealand; 2 Department of Communications and New Media, National University of Singapore, Singapore 119077, Singapore; somrita987@gmail.com

**Keywords:** COVID-19 vaccine intention, vaccine hesitancy, theory of planned behaviour, vaccine attitudes, social norms, efficacy, longitudinal data, behavior change, vaccination campaigns, New Zealand

## Abstract

While public intentions to get a COVID-19 vaccine have been shifting around the world, few studies track factors that help us understand and improve COVID-19 vaccine uptake. This study focuses on identifying changing public intentions to get a COVID-19 vaccine in New Zealand, a country that has been largely successful in containing the pandemic but risks new outbreaks as less than 20% of the population is fully vaccinated by August 2021. Data on COVID-19 intentions were collected just after the vaccine approval and rollout targeting old-age groups in February 2021 and then before the general public rollout in May 2021 (*n* = 650, 60% reinterview response rate). Results show that intention to get a COVID-19 vaccine increased in three months and was the highest in the last one year. Consistent with the Theory of Planned Behaviour, attitudes and efficacy beliefs were significantly associated with COVID-19 vaccine intentions, in the cross-sectional as well as longitudinal analyses. Findings highlight the persisting influence of attitudes, efficacy beliefs, and past intentions on future decision-making process to get a COVID-19 vaccine. Future research opportunities to understand vaccine intentions and improve public vaccine uptake are highlighted.

## 1. Introduction

Public intentions to get a COVID-19 vaccine have been changing around the world [1,2,3,4]. For example, public willingness to vaccinate against COVID-19 has declined from 87% in April to 79% in August 2020 in Australia [5], from 71% in April to 54% in October 2020 in the US [6], and from 92% in March to 87% in December 2020 in China [4]. Previous research highlights that limited community spread of COVID-19, perceived low risk of getting infected, distrust with government and experts, and vaccine side effects has led to the decline in COVID-19 vaccination intentions [2,7,8]. Following the vaccine rollout in several countries in 2021, public willingness to get a COVID-19 has increased, however [9].

New Zealand is one of the few countries in the world that has largely evaded the pandemic [10] and was consistently ranked as the best country in the world that was responding to COVID-19 [11]. Yet, a slow vaccine rollout with only 20% of the population fully vaccinated by August 2021, combined with the need to get a large proportion of the population vaccinated in anticipation of the Delta variant, poses a risk to the elimination strategy that New Zealand has so far successfully applied [12,13]. To safely open the borders, health experts say there needs to be almost universal COVID-19 vaccination rates—a tough challenge given a declining rate of vaccination in general [14], and COVID-19 vaccine hesitancy and scepticism in particular [15].

This research explores the changing intentions to get a COVID-19 vaccine in New Zealand using a longitudinal study based on the Theory of Planned Behaviour (TPB) framework [16]. TPB posits those attitudes, subjective norms, and perceived behaviour control are key antecedents of behavioural intention, which is a proximate predictor of behaviour. In addition, perceived behavioural control is expected to have a direct association with behaviours in so far as the target behaviours are within one’s realm of influence. Finally, past behaviour is considered as an additional predictor of intention and behaviour. A number of empirical studies and meta-analytic reviews, across a variety of personal health behaviours, provide credence to these theory predictions [17,18,19]. Generally, perceived behaviour control is considered as reflected in self-efficacy or beliefs that one has the capabilities to perform directed health behaviours [20,21]. When both perceived control and self-efficacy variables are tested, self-efficacy appears to be more strongly associated with health behaviours [17,21].

However, the TPB is not without limitations. First, a majority of evidence is based on cross-sectional surveys, where the intention-behaviour gap is less visible than in longitudinal studies [19,22,23]. For example, the TPB constructs explain about half of variance in intentions but only a minor variance in behaviours in a meta-analysis of TPB to predict nutrition-related behaviours in youth [19]. However, limited number of studies have been conducted using TPB in longitudinal analysis to identify vaccination intention [24,25] and a majority of reports with few exceptions used cross-sectional TPB framework [26,27,28].

Second, the few longitudinal studies on COVID-19 vaccine intentions have evaluated all TPB constructs: while some focus on attitudes alone without also encapsulating social norms and efficacy [3,6], while others on perceived risk of the disease [22]. Several studies have focussed on, for example, vaccine efficacy communication (how effective are the vaccines) [29,30,31] rather than self-efficacy, even as self-efficacy has been found to be an important predictor of intentions and behaviours [32].

Furthermore, it is not clear if attitudes, norms, and efficacy beliefs association with intentions persist over time. Several studies conducted in Netherlands on HPV vaccination show that the association between attitudes, norms, and efficacy beliefs with behavioural uptake is mediated through intentions [24,33,34], consistent with research in other health domains. In contrast, Wu et al., [25] conducted a longitudinal study of Chinese parents in Hong Kong and found that parental positive attitude, norm, and intention—but not negative attitudes and behaviour control—were significant predictors of their child’s influenza vaccination only among parents who had previously vaccinated their children. Among parents who had not previously vaccinated their children, only intention was predictor of future influenza vaccine uptake. They asserted that TPB is less predictive of new health-related behaviour than predicting repeated behaviours. A recent study longitudinally tested TPB on COVID-19 safety behaviours (but not vaccine intentions) and found that baseline attitudes, norms, and perceived behaviour control for social distancing were all associated with baseline intentions to social distance, which in turn were significantly associated with social distancing behaviour at follow-up in 3 months [22].

This research tests if attitudes, social norms, and efficacy beliefs are significantly associated with COVID-19 vaccine intentions at the baseline and after three months using a national sample survey in New Zealand. This manuscript contributes to research that clarifies the role of TPB constructs on intentions over time as previous studies have found mixed evidence. Findings of this study will help develop TPB and will be useful for the New Zealand government health authorities to improve COVID-19 vaccine uptake.

## 2. Materials and Methods

Data for this study was collected using nationally representative online panels of Qualtrics and affiliates in New Zealand in between February-March 2021 (*n* = 1083), when the vaccine approval rollout was just announced and was targeted towards high-risk older age groups, and then again between April-May 2021, shortly before the vaccination programme was to open for the general public. In total, 650 respondents completed both the surveys, and the reinterview response rate was 60%. Compared to the complete sample in March 2021, the follow-up panel survey composed of slightly more older respondents (66 years and above), European New Zealanders, and fewer Māori. The sample composition was similar on gender, education, and annual income between the two waves (See Table 1).

Qualtrics and its affiliates maintain nationally representative active panels composed of over ~350,000 respondents in New Zealand. The respondents are recruited using a variety of media channels and they receive gift cards or sweepstakes entries for larger prizes to participate. Hard-to-reach population receive additional compensation for taking and for recruiting others to do a survey, ensuring a good representative sample. Nevertheless, the population of our online sample were slightly more likely to be educated, and fewer respondents who self-identify with Pasifika ethnicity. Overall, the demographics align well with national Census estimates on gender, age, and ethnicity proportions.

Ethics approval was filed at the (Removed for peer-review) University’s human research review board (ethics number: removed for peer-review) and the study was determined to be low risk and exempt from a full review. Participants provided informed consent after reading brief aims of the survey—namely, to understand public opinion about current issues facing the country and the world. The average time to complete the survey was 22 min.

### 2.1. Measures

The primary outcome measure was COVID-19 vaccination intention, adopted from previous studies [35]. Specifically, the respondents were asked, “When a coronavirus (COVID-19) vaccine becomes available—Would you accept the vaccine for yourself?” The responses were measured on a four-point scale from “Yes, definitely” (1), “Unsure, but leaning towards Yes” (2), “Unsure, but leaning towards No” (3), and “No, definitely not” (4). Higher values indicate higher COVID-19 vaccine hesitancy. The mean vaccine hesitancy was higher in March (*M* = 1.68, *SD* = 0.98) compared to May 2021 (*M* = 1.59, *SD* = 0.96).

COVID-19 vaccine attitudes were assessed using measures adapted from previous studies [36]. Respondents rated, “To what extent do you feel that getting a COVID-19 vaccine will be….” on a scale consisting of 1–7 semantic differential items (bad/good, unpleasant/pleasant, undesirable/desirable, harmful/beneficial, worthless/valuable, ineffective/effective, unsafe/safe) (*α* = 0.96, Kaiser–Meyer–Olkin measure = 0.94, Bartlett’s test of sphericity (*χ*^2^ (21) = 5465.02, *p* < 0.001, total variance explained by the one factor was 89%). Higher scores indicate more favourable attitudes towards a COVID-19 vaccine.

Social norms were measured using three items following previous studies [37] and were prefaced by asking “How much do you agree or disagree with the following statements….” The responses ranged from “strongly disagree” (1) to “strongly agree” (5) with “neither” as a mid-point (3). The questions were, “Most of my family members and friends will take a COVID-19 vaccine when available” (*M* = 3.97, *SD* = 1.10), “Most people who are important to me would approve of my getting a COVID-19 vaccine when available” (*M* = 4.10, *SD* = 1.06), and “Doctors would think that I should get a COVID-19 vaccine when available” (*M* = 4.19, *SD* = 0.98). The three items were treated as descriptive norm, subjective norm, and injunctive norm.

Self-efficacy to get a vaccine was measured by asking respondents, “I am confident that I will get a COVID-19 vaccine as soon as it is available in my area” (*M* = 3.77, *SD* = 1.32) on a similar 5-point scale as mentioned above.

Demographic variables collected included gender, age, education, income, ethnicity, and ideological orientation (5-point scale from “very conservative” to “very liberal”). See Table 1 for sample demographics between the two surveys.

### 2.2. Analysis

Analysis began in a stepwise fashion, checking individual variables, correlation between important constructs in the study and then building hierarchical regression models to assess the unique contribution of each group of variables in explaining the outcome variable of [38] vaccine intentions measured three months later in May 2021. All data was analysed using SPSS, version 27.0. Similar to previous studies, TPB constructs were treated as continuous variables. As suggested by a reviewer, we re-examined the relationships using a multinomial logistic regression, comparing those who would ‘definitely’ get a vaccine to other responses, and treating other key constructs as categorical variables. The results do not change, and we therefore choose to report a simpler model as below (see Supplementary Tables S1 and S2).

As suggested by a reviewer, missing data analysis was conducted to account for attrition. We used SPSS (ver. 27) software for multiple imputation, which uses a iterative Markov chain Monte Carlo (MCMC) method known as fully conditional specification. The fully conditional specification (FCS) method was suitable because it uses all other variables in the model—in this case, attitudes, norms, efficacy, and intention from wave 1—as predictors and then imputes missing values for the outcome variable, vaccine intentions in wave 2. The multiple regression results across the five imputed models and the pooled results are consistent with the findings of the complete data analysis reported in this study (see Supplementary Table S3).

## 3. Results

In March 2021, 60.1% of the respondents said they will ‘definitely’ take a COVID-19 vaccine when available, which jumped to 66.6% in May 2021. There was a decline among those who were “unsure but leaning towards Yes” from 19.1% in March to 15.8% in May as well as among those who were “unsure but leaning towards No” from 11.7% in March to 9.5% in May. Those who said “definitely” no to get a vaccine remained stable, from 8.6% in March to 8% in May. In other words, while there was a shift towards greater intention to take a COVID-19 vaccine, a minority continued to be opposed to a vaccine.

The correlations table (Table 2) shows strong positive association between vaccine intentions in March and May (*r* = 0.76, *p* < 0.001), indicating that intentions were largely stable across time. Intentions in March and May were also strongly associated with attitudes, social norms, and efficacy beliefs, with more positive attitudes towards vaccine, higher degree of positive social norms to get a vaccine, and efficacy to get a COVID-19 vaccine associated negatively with vaccine hesitancy at both time-periods.

The first hierarchal regression model included only demographic variables and explained only 4% of variance in intentions to get a COVID-19 vaccine in May 2021. Women compared to men, younger, lower educated, low income, and those with conservative ideology were less likely to get a COVID-19 vaccine compared to their respective others (See Table 3). There was no difference in ethnicity, but Māori were slightly less likely to get a vaccine compared to Asian and others (*B* = 0.28, *SE* = 0.15, *p* = 0.06).

In the second model, attitudes, social norms, and efficacy explained an additional 51% of variance in intentions to get a COVID-19 vaccine. However, none of the demographic variables remained significant anymore in the model.

More favourable attitudes were negatively associated with vaccine hesitancy. A similar negative association was found between efficacy beliefs and vaccine hesitancy. In other words, the higher the favourable values towards COVID-19 vaccine, the higher the efficacy beliefs, the higher are the intentions to get a COVID-19 vaccine three months later in May 2021. There was no significant association between descriptive, subjective, and injunctive norms, with vaccine hesitancy.

Adding baseline vaccine intention explained additional 6% variance in intentions to vaccinate against COVID-19 in May. In the presence of baseline vaccine intentions, baseline attitudes and efficacy beliefs remained significantly associated with vaccine intentions in May 2021, indicating that even after controlling previous intentions, these attitudes and efficacy beliefs have a unique and persisting influence in shaping vaccination choices in future.

A similar hierarchical regression model, with baseline vaccine intentions collected in March 2021 as the outcome variable was tested. Similar to the above findings, while demographic variables accounted for only 6% of variance, TPB constructs of attitudes, social norms, and efficacy beliefs accounted for 74% of variance in vaccine intentions at baseline. In addition to attitudes and efficacy beliefs, social norms were significantly associated with baseline intentions. While descriptive and subjective norms were significantly associated with higher intentions to get a vaccine, higher injunctive norms were associated with increased vaccine hesitancy, contrary to expectations.

The consistency between cross-sectional and longitudinal findings suggest that attitudes and efficacy beliefs largely account for differences in COVID-19 vaccination intentions but that previous intentions also shape future decision-making process about a COVID-19 vaccine.

## 4. Discussion

Public acceptance of a COVID-19 vaccine has been changing around the world and previous studies generally show a decline [3,4,5], while some show an uptick [9]. To examine changes in New Zealanders’ intentions to get a COVID-19 vaccine, two consecutive surveys were conducted just after the vaccine approval and rollout for high-risk groups in February–March 2021 and in April–May 2021, just before vaccine rollout for the general public. Similar to the Ministry of Health funded surveys [15], public intentions to get a vaccine was at its highest in May 2021 in the last one year. In contrast to other studies that have reported a decline in the US, Australia and China [4,5,6], there is consensus across multiple opinion surveys in New Zealand [15,39] of an increase in vaccine intention. Yet, about one in five New Zealanders has remained vaccine hesitant or sceptic despite a safe rollout and the increased risks due to Delta variant across the world, which health experts say requires even higher proportion, between 80–90% or more, of the population to be vaccinated [13].

Consistent with the TPB framework, attitudes and self-efficacy was significantly associated with COVID-19 vaccine intention. The three constructs of TPB explained 74% of variance in COVID-19 vaccine intentions, when measured simultaneously, and 55% of variance in vaccine intentions three months later.

Attitudes provide easily accessible information to form behavioural intentions and the findings of this study show that these associations continue to persist over time. Attitudes have been found to be strongly associated with intentions, as studies in vaccine intentions [40] in general, and COVID-19 prevention or mitigation intentions [1,6] in particular, attest. Given the importance of attitudes in predicting intentions and behaviour, future research and interventions should focus on how changing attitudes can help increase public enthusiasm for vaccination.

Efficacy beliefs was one of the strongest correlates of COVID-19 vaccine intention, consistent with theoretical postulates and empirical evidence [21,41]. Previous COVID-19 vaccine intention studies have largely ignored factoring in the key variable of efficacy, instead focussing on vaccine efficacy communication [29,30]. While it is important to communicate how effective COVID-19 vaccines are in preventing severe illness and mortality, the findings of this study highlight the need to make self-efficacy part of the strategy to communicate about COVID-19 vaccines. In particular, messages about ease, control, and ownership may help improve public enthusiasm for vaccines [42].

Further, while baseline measure of social norms—descriptive, subjective, and injunctive—were significantly associated with baseline intentions but not intentions after three months. It is not clear why, and it is possible that the effect of social norms on intentions and subsequent behaviour is not as persistent as attitudes and efficacy beliefs. Other studies also show no association between social norms in encouraging vaccine behaviour, arguing instead that attitudes are better predictor of public-facing health behaviours rather than private behaviours such as vaccination [17,40]. At least one study in the UK shows that communicating descriptive COVID-19 vaccination norms to young people, even by other young people, does not increase vaccine intentions any more than a standard government notification on vaccines [43]. It is also possible that the association between social norms and vaccine intentions is mediated by other variables of TPB [41]. These mixed findings highlight the need for more research on how social norms align with COVID-19 vaccine intentions and future vaccine uptake.

So far, only a few research articles have been published on New Zealand public intentions to get a COVID-19 vaccine [39,44]. These, however, are cross-sectional surveys, informing us little about how public willingness to get a vaccine is shifting along with increased vaccine access to the general public. Consistent with public opinion surveys in other countries such as the US and UK, with increasing vaccine access, vaccine hesitancy has declined in New Zealand [9]. Nevertheless, in some countries such as UK and US, vaccination rates had a sharp uptick after the initial vaccine rollout but then plateaued at about 70% of the public vaccinated at least with one dose of a COVID-19 vaccine [45,46,47].

This will be the primary challenge for the New Zealand government when it rolls out the vaccination programme for all adults in the country: while it will probably reach 60–70% of the population relatively quickly, it will struggle to get above that figure. The rise in new cases and deaths in the US are primary among the unvaccinated, indicating the need to improve high levels of COVID-19 vaccine uptake to protect self and to achieve population immunity.

The study is not without limitations. While online samples are largely representative, they still tend to be over-represented by higher education and income levels. A larger, more representative sample may provide slightly different estimates than reported above. At both times of the survey, vaccination intentions were self-reported, and it is important to follow-up on actual behaviour, particularly as few studies report intention-behaviour gap [22]. Similarly, other variables of perceived susceptibility and severity from Health Belief Model should be evaluated for a more comprehensive model [1,4,27]. The associations between social norms and vaccine intentions—tested using multinomial logistic models and multiple imputation models—were inconsistent and weak at best. A simpler analysis is reported in the study as findings that are consistent across different treatment of variables and analytical techniques are more robust. Future researchers should pay more attention to report these details to assess association as theoretical postulated.

It is important to note, however, that intention to get a vaccine declined between a severe epidemic phase and a well-contained phase in China [4] and in Australia [5], indicating that public judgements change over time and that external risks may not always be a prime motivation for people to get a COVID-19 vaccine. Future research should investigate how changing severity of the disease spread influence vaccine intentions. Recent research shows that vaccine hesitant and unvaccinated are much less worried about the coronavirus, the Delta variant, and have less confidence in the safety and effectiveness of the vaccines compared to those who are vaccinated [48]. Future research should also survey medical staff, social workers, and other frontline healthcare staff on vaccination intentions. The influence of media sources and trust on intentions to get a COVID-19 vaccine and to encourage others to get one should also be investigated [28]. As booster shots are likely in future due to reduced efficacy of the vaccine against new variants, it is important to track changing public opinion on getting a vaccine now and planning for one in future.

## 5. Conclusions

The rise of new, more infectious and probably more deadly COVID-19 variants has led governments to push for higher vaccination rates. Yet, previous studies show a decline in intention to vaccinate. Even after an initial uptick following vaccine rollout, public enthusiasm for vaccination stalls after reaching 60–70% of the population. The findings of this study illustrate that the association between attitudes and efficacy beliefs persist over time. At the same time, previous intentions also play a key role in future decision-making processes about getting a COVID-19 vaccine or refusing one. Campaigns that aim to help change public attitudes, social norms, and self-efficacy beliefs are likely to have short-term and long-term impact on vaccination uptake.

## Figures and Tables

**Table 1:** Demographic characteristics of the longitudinal sample.

Variable		(Full Sample, March 2021)		(Panel, May 2021)	
		*n* = 1083	%	*n* = 650	%
Gender	Female	552	51	324	50
	Male	531	49	326	50
Age	18–25	155	14	53	8
	26–35	215	20	115	18
	36–45	174	16	81	13
	46–55	193	18	138	21
	56–65	162	15	110	17
	66 and above	184	17	153	24
Education	No qualification	87	8	45	7
	School qualification	286	26	177	27
	Tertiary diplomas/Certificates	335	31	199	31
	Bachelor’s degree or higher	375	35	229	35
Ethnicity	European New Zealander	689	64	457	70
	Māori	175	16	69	11
	Pasifika	43	4	25	4
	Asian or Another	176	16	99	15
Annual income	Less than $19,999	208	19	121	19
	$20,000 to $39,999	261	24	164	25
	$40,000 to $59,999	197	18	124	19
	$60,000 to $79,999	184	17	110	17
	$80,000 to $99,999	94	9	63	10
	$100,000 to $119,999	66	6	30	5
	$120,000 or above	66	6	34	5

**Table 2:** Correlations between key constructs.

	Variable Name	1	2	3	4	5	6	7
1	COVID-19 vaccine intention (May 2021)	1						
2	COVID-19 vaccine intention (March 2021)	0.763 **	1					
3	Attitudes towards COVID-19 vaccine	−0.737 **	−0.648 **	1				
4	Descriptive norm	−0.692 **	−0.583 **	0.603 **	1			
5	Subjective norm	−0.681 **	−0.561 **	0.598 **	0.820 **	1		
6	Injunctive norm	−0.535 **	−0.469 **	0.481 **	0.629 **	0.665 **	1	
7	Self-efficacy	−0.795 **	−0.699 **	0.673 **	0.696 **	0.681 **	0.631 **	1

Note: ** *p* < 0.001. Higher values of COVID-19 vaccine intention represent higher vaccine hesitancy to get a vaccine.

**Table 3:** Hierarchical multiple regression analysis with baseline and follow-up COVID-19 vaccine intentions as outcome variables.

	Intention to Get a COVID-19 Vaccine (March 2021) ^a^				Intention to Get a COVID-19 Vaccine (May 2021) ^b^					
	Model 1		Model 2		Model 3		Model 4		Model 5	
	*B (SE)*	*β*	*B (SE)*	*β*	*B (SE)*	*β*	*B (SE)*	*β*	*B (SE)*	*β*
(Constant)	2.75(0.25)		4.72(0.15)		2.34(0.25)		3.97(0.19)		1.8(0.28)	
Female	0.16(0.08)	0.08 *	0.11(0.04)	0.06 *	0.15(0.08)	0.08	0.11(0.05)	0.06	0.06(0.05)	0.03
Age	−0.08(0.03)	−0.13 **	0.02(0.01)	0.03	−0.08(0.02)	−0.14 ***	0(0.02)	0.00	−0.01(0.02)	−0.01
Education	−0.09(0.05)	−0.09 *	−0.01(0.02)	−0.01	−0.11(0.04)	−0.11 *	−0.04(0.03)	−0.04	−0.04(0.03)	−0.04
Income	−0.03(0.03)	−0.05	−0.04(0.01)	−0.07 **	0(0.03)	0.00	−0.01(0.02)	−0.01	0.01(0.02)	0.02
Ideology	−0.16(0.04)	−0.16 ***	0.01(0.02)	0.01	−0.1(0.04)	−0.10 *	0.05(0.03)	0.05	0.04(0.03)	0.04
Ethnicity										
European NZ	−0.03(0.11)	−0.02	−0.11(0.06)	−0.05	0.11(0.11)	0.05	0.03(0.08)	0.02	0.09(0.07)	0.04
Māori	0.27(0.16)	0.08	0(0.08)	0.00	0.28(0.15)	0.09	0.05(0.11)	0.02	0.06(0.1)	0.02
Pasifika	0.21(0.22)	0.04	−0.22(0.12)	−0.04	0.2(0.21)	0.04	−0.18(0.15)	−0.04	−0.07(0.14)	−0.02
Vaccine attitudes			−0.2(0.02)	−0.31 ***			−0.19(0.02)	−0.31 ***	−0.1(0.02)	−0.16 ***
Social norms										
Descriptive norm			−0.1(0.03)	−0.11 **			−0.07(0.04)	−0.08	−0.03(0.04)	−0.03
Subjective norm			−0.13(0.04)	−0.14 ***			−0.04(0.05)	−0.04	0.02(0.04)	0.02
Injunctive norm			0.06(0.03)	0.06 *			0.02(0.04)	0.02	−0.01(0.03)	−0.01
Self-efficacy			−0.34(0.03)	−0.45 ***			−0.3(0.03)	−0.42 ***	−0.15(0.03)	−0.20 ***
COVID-19 vaccine intention (March 2021)									0.46(0.05)	0.48 ***
∆*R*^2^	0.06		0.74		0.04		0.55		0.61	

Note: *n* = 650. Female compared to male. Asians were the reference category for ethnicity categories in the table. Higher values of COVID-19 vaccine intention represent higher vaccine hesitancy to get a vaccine. *B* = Unstandardized regression coefficient, *SE* = Standard Error, and *β* = Standardized regression coefficient. **^a^** Hierarchical multiple regression predicting vaccine intentions from data collected during wave 1. Model 1 outcome variable is vaccine intentions at wave 1 and predictor variables are demographic variables only. Model 2 outcome variable is vaccine intentions at wave 1 consisting of demographic variables and predictor variables collected at wave 1. **^b^** Hierarchical multiple regression predicting vaccine intentions in wave 2 using predictor variables collected during wave 1. Model 3 outcome variable is vaccine intentions in wave 2 and predictor variables are demographic variables collected during wave 1. Model 4 and 5 represent models predicting vaccine intentions in wave 2 using demographic variables and predictor variables collected during wave 1. * *p* < 0.05, **p < 0.01, *** *p* < 0.001.

## Data Availability

The data presented in this study are available on request from the corresponding author. The data are not publicly available due to on-going research project.

## Supplementary Materials

The following are available online at https://www.mdpi.com/article/10.3390/vaccines9101132/s1, S1: Likelihood Ratio Tests for multinominal logistic regression with ’Yes, Definitely’ get a vaccine as reference category and using all other variables categorical, Table S2: Multinominal logistic regression with ’Yes, Definitely’ get a vaccine as reference category and using all other variables categorical; Table S3: Multiple regression predicting intention to get a COVID-19 vaccine in wave 2 in the 5 imputed datasets and pooled results.
