# Supplementary material for: The Role of Attitudes, Norms, and Efficacy on Shifting COVID-19 Vaccine Intentions: A Longitudinal Study of COVID-19 Vaccination Intentions in New Zealand
Source: Vaccines (Basel). 2021 Oct 4;9(10):1132. doi: 10.3390/vaccines9101132 (PMC8570329; doi:10.3390/vaccines9101132)
Supplement: Supplementary file 1 [file vaccines-09-01132-s001.zip › vaccines-1364463-supplementary.pdf]

Supplementary Table S1. Likelihood Ratio Tests for multinominal logistic regression with 'Yes, Definitely' get a vaccine as reference category and using all other variables categorical.

Likelihood Ratio Tests						
Effect	Model Fitting Criteria			Likelihood Ratio Tests		
	AIC of Reduced Model	BIC of Reduced Model	-2 Log Likelihood of Reduced Model	Chi-Square	df	Sig.
Intercept	889.901	1400.101	661.901	0	0	.
Gender	886.587	1383.36	664.587	2.685	3	0.443
Age	867.269	1310.337	669.269	7.367	15	0.947
Education	875.418	1345.339	665.418	3.517	9	0.94
Income	876.63	1319.698	678.63	16.729	15	0.335
Ethnicity	878.713	1348.633	668.713	6.811	9	0.657
Attitudes towards COVID-19 vaccine Wave 1	901.332	1398.105	679.332	17.431	3	0.001
Descriptive norm	876.859	1333.353	672.859	10.957	12	0.533
Subjective norm	878.559	1335.053	674.559	12.658	12	0.394
Injunctive norm	875.395	1331.889	671.395	9.494	12	0.66
Self-efficacy	886.573	1343.067	682.573	20.671	12	0.055
COVID-19 vaccine intention (March 2021)	948.332	1418.253	738.332	76.431	9	0
Pseudo R-Square						
Cox and Snell	0.617					
Nagelkerke	0.716					
McFadden	0.485					

Supplementary Table S2. Multinominal logistic regression with 'Yes, Definitely' get a vaccine as reference category and using all other variables categorical.

	Unsure, but leaning towards YES				Unsure, but leaning towards NO				No, definitely not			
	Exp(B)	95% CI		Sig.	Exp(B)	95% CI		Sig.	Exp(B)	95% CI		Sig.
Male	0.91	0.50	1.67	0.77	0.53	0.22	1.30	0.17	0.50	0.17	1.48	0.21
Female (reference)												
18-25	1.89	0.58	6.15	0.29	1.92	0.28	13.01	0.51	2.32	0.24	22.24	0.47
26-35	1.77	0.64	4.88	0.27	1.69	0.35	8.23	0.52	1.57	0.23	10.65	0.65
36-45	2.24	0.76	6.60	0.14	3.03	0.60	15.43	0.18	5.16	0.75	35.63	0.10
46-55	1.39	0.53	3.60	0.50	1.50	0.33	6.73	0.60	2.60	0.44	15.52	0.30
56-65	1.02	0.36	2.87	0.97	1.68	0.37	7.64	0.50	3.25	0.54	19.52	0.20
66 and above (reference)	.	.	.	.	.	.	.	.	.	.	.	.
No qualification	1.31	0.38	4.51	0.67	2.45	0.45	13.29	0.30	2.60	0.33	20.73	0.37
School qualification	1.37	0.59	3.18	0.47	1.05	0.30	3.66	0.94	1.97	0.43	9.03	0.39
Tertiary diplomas/Certificates	1.15	0.53	2.48	0.72	1.08	0.36	3.27	0.90	0.98	0.22	4.33	0.98
Bachelor’s degree or higher (reference)	.	.	.	.	.	.	.	.	.	.	.	.
Less than $19,999	1.26	0.40	4.00	0.70	0.52	0.10	2.65	0.43	0.55	0.08	3.88	0.55
$20,000 to $39,999	1.46	0.47	4.58	0.52	0.73	0.14	3.87	0.71	1.32	0.20	8.80	0.78
$40,000 to $59,999	1.17	0.38	3.57	0.79	1.55	0.33	7.36	0.58	0.77	0.12	5.07	0.79
$60,000 to $79,999	2.21	0.75	6.53	0.15	0.86	0.18	4.27	0.86	3.41	0.53	22.01	0.20
$80,000 to $99,999	2.04	0.57	7.30	0.27	2.87	0.42	19.81	0.28	0.01	0.00	0.01	1.00
$100,000 and over (reference)	.	.	.	.	.	.	.	.	.	.	.	.
European New Zealander	1.91	0.81	4.50	0.14	1.44	0.41	5.10	0.58	3.62	0.65	20.25	0.14
Māori	2.00	0.66	6.12	0.22	1.44	0.28	7.33	0.66	1.51	0.16	14.24	0.72
Pasifika	1.94	0.42	9.04	0.40	0.84	0.10	7.10	0.87	0.48	0.03	8.06	0.61
Asian or Another (reference)	.	.	.	.	.	.	.	.	.	.	.	.
Attitudes towards COVID-19 vaccine Wave 1	0.67	0.51	0.88	0.00	0.47	0.32	0.69	0.00	0.62	0.40	0.98	0.04
Descriptive norm (1= strongly disagree)	7.98	0.19	327.81	0.27	5.91	0.07	498.96	0.43	20.61	0.31	1371.83	0.16
	2.32	0.45	11.85	0.31	1.14	0.10	12.98	0.91	1.90	0.15	24.43	0.62
	2.45	0.72	8.35	0.15	1.71	0.21	13.88	0.61	0.37	0.03	4.01	0.41
	1.89	0.67	5.32	0.23	1.32	0.20	8.45	0.77	0.65	0.08	5.15	0.69
Descriptive norm (5= strongly agree), reference	.	.	.	.	.	.	.	.	.	.	.	.
Subjective norm (1 = Strongly disagree)	0.83	0.03	27.44	0.92	0.01	0.00	0.67	0.03	0.04	0.00	3.57	0.16
	1.08	0.21	5.55	0.93	0.25	0.03	2.22	0.21	0.90	0.07	12.26	0.94
	1.98	0.55	7.07	0.30	0.44	0.07	2.89	0.39	4.84	0.49	47.87	0.18
	1.55	0.57	4.23	0.39	0.60	0.12	2.94	0.53	1.65	0.18	15.28	0.66

Subjective norm (5 = Strongly agree), reference	.	.	.	.	.	.	.	.	.	.	.	.
Injunctive norm (1 = Strongly disagree)	0.01	0.01	0.01	.	0.50	0.02	14.45	0.69	0.98	0.07	13.97	0.99
	0.45	0.08	2.45	0.35	0.44	0.05	3.86	0.46	0.55	0.04	7.08	0.64
	0.84	0.31	2.30	0.74	2.28	0.52	9.92	0.27	1.08	0.18	6.34	0.93
	0.73	0.31	1.75	0.48	1.42	0.37	5.50	0.61	0.76	0.14	4.08	0.75
Injunctive norm (5= Strongly agree), reference	.	.	.	.	.	.	.	.	.	.	.	.
Self-efficacy (1 = Strongly disagree)	10.52	1.50	73.69	0.02	38.60	2.10	711.08	0.01	18.45	1.40	242.64	0.03
	9.60	2.42	38.16	0.00	15.50	1.05	229.07	0.05	3.87	0.34	44.41	0.28
	4.15	1.27	13.55	0.02	7.17	0.56	92.54	0.13	2.53	0.23	27.96	0.45
	3.31	1.20	9.10	0.02	3.45	0.30	39.70	0.32	2.65	0.29	24.10	0.39
Self-efficacy (5 = Strongly agree), reference	.	.	.	.	.	.	.	.	.	.	.	.
COVID-19 vaccine intention (March 2021)												
Yes, definitely	4.83	0.45	51.94	0.19	0.09	0.01	1.08	0.06	0.03	0.00	0.47	0.01
Unsure, but leaning towards Yes	24.61	2.51	241.38	0.01	0.51	0.07	3.68	0.50	0.10	0.01	1.03	0.05
Unsure, but leaning towards No	17.86	1.86	171.16	0.01	5.83	0.95	35.91	0.06	0.40	0.06	2.96	0.37
No, definitely not (reference)	.	.	.	.	.	.	.	.	.	.	.	.

Note. *N* = 650. The reference category is 'Yes, definitely'.

Supplementary Table S3. Multiple regression predicting intention to get a COVID-19 vaccine in wave 2 in the 5 imputed datasets and pooled results.

	Model 1			Model 2			Model 3			Model 4			Model 5			Pooled		
	B	*SE*	Sig.	*B*	*SE*	Sig.	*B*	*SE*	Sig.	*B*	*SE*	Sig.	*B*	*SE*	Sig.	*B*	*SE*	Sig.
(Constant)	1.81	0.21	0.00	1.80	0.22	0.00	1.53	0.21	0.00	1.61	0.22	0.00	1.37	0.22	0.00	1.62	0.30	0.00
Vaccine attitudes	-0.11	0.02	0.00	-0.09	0.02	0.00	-0.07	0.02	0.00	-0.08	0.02	0.00	-0.08	0.02	0.00	-0.09	0.03	0.00
Descriptive norm	-0.04	0.03	0.17	0.04	0.03	0.17	-0.04	0.03	0.14	-0.04	0.03	0.17	-0.04	0.03	0.21	-0.02	0.05	0.64
Subjective norm	0.06	0.03	0.05	0.03	0.03	0.43	0.08	0.03	0.01	0.09	0.03	0.00	0.11	0.03	0.00	0.07	0.05	0.14
Injunctive norm	0.00	0.03	0.97	0.02	0.03	0.52	0.02	0.03	0.56	-0.10	0.03	0.00	-0.03	0.03	0.31	-0.02	0.06	0.76
Self-efficacy	-0.14	0.03	0.00	-0.22	0.03	0.00	-0.17	0.03	0.00	-0.07	0.03	0.01	-0.12	0.03	0.00	-0.14	0.06	0.07
COVID-19 vaccine intention (March 2021)	0.46	0.03	0.00	0.44	0.04	0.00	0.47	0.04	0.00	0.54	0.04	0.00	0.48	0.04	0.00	0.48	0.06	0.00
ΔR^2	0.604			0.560			0.563			0.568			0.515					

Note: N=1083. *B*=Unadjusted regression coefficient, *SE* = Standard Error, Sig. = Significance. The models above are adjusted for gender, age, education, income, ethnicity, and political ideology. Multiple imputation was used to generate 5 imputed datasets (*n=6498*). Imputation models included other wave 1 variables used in the analysis such as attitudes, norms, efficacy, and intentions to get a COVID-19 vaccine.
